# Supplementary material for: Surface Microbiome Profiling of Dental Elevators Using Third‐Generation Sequencing: Implications for Infection Control in Dental Practice
Source: Microbiologyopen. 2025 Dec 8;14(6):e70178. doi: 10.1002/mbo3.70178 (PMC12685768; doi:10.1002/mbo3.70178)
Supplement: Supplementary file 1 — customer_backup. [file MBO3-14-e70178-s001.zip › customer_backup/customer_backup/taxa_summary/krona/groups/treat/M14.Krona.html]

Javascript must be enabled to view this page.

members
magnitude
magnitudeUnassigned

M14.krona

59664

59663

51

0

0

0

0

0

0

0

0

0

0

0

0

0

0

0

51

51

51

51

0

27

0

0

0

23

0

0

1

0

0

0

0

0

0

0

0

0

0

0

0

0

0

44

12

0

0

0

0

5

5

2

2

2

1

1

0

0

1

1

0

0

7

3

3

3

1

1

1

0

0

0

0

0

0

0

0

0

0

3

2

2

0

0

0

0

1

1

0

0

0

0

0

0

32

30

0

0

0

30

30

30

2

0

0

0

0

0

0

0

0

0

0

0

0

2

2

2

435

323

323

311

311

311

12

8

8

0

4

3

1

0

0

0

0

1

0

0

0

0

1

0

0

0

0

0

0

0

0

0

0

0

0

0

1

0

0

0

0

0

0

0

0

0

0

0

0

0

1

1

0

0

0

0

0

0

0

0

0

0

0

0

0

0

0

110

110

42

0

0

0

0

1

1

0

0

40

40

1

1

0

0

0

0

0

0

0

0

0

0

0

68

0

0

0

0

0

68

68

1

1

1

0

0

1

1

0

0

0

0

0

0

0

0

0

0

0

0

0

0

0

0

0

0

0

0

0

0

0

0

0

0

0

0

0

0

0

0

0

0

0

0

0

0

0

0

0

0

0

0

0

0

8

8

8

0

0

0

0

0

0

8

8

8

0

0

0

0

0

0

532

203

203

203

0

0

58

58

130

0

130

15

15

0

7

7

7

7

7

322

322

322

75

75

0

241

0

0

189

0

52

6

6

0

0

0

255

63

63

0

0

0

63

63

63

0

0

0

0

0

0

0

0

0

0

0

0

0

0

0

0

192

75

75

0

0

0

0

75

0

64

11

0

0

0

0

0

0

0

0

0

0

0

0

0

0

104

43

0

0

0

0

43

31

12

0

0

0

0

0

0

0

54

0

0

0

54

0

54

0

0

7

7

1

6

13

13

0

0

0

0

0

0

1

0

1

12

12

0

0

0

0

0

20113

12285

0

0

0

0

575

127

127

127

349

33

25

8

37

28

9

4

4

275

0

275

0

99

99

99

209

209

159

0

159

42

42

8

8

17

0

0

0

5

0

0

0

0

0

0

5

5

12

12

12

93

93

92

0

0

92

1

0

0

0

1

24

24

24

24

0

0

0

6803

6803

1

1

266

61

16

88

1

100

0

0

56

56

26

26

200

200

19

19

0

0

0

0

12

0

0

0

6

0

0

6

1

1

886

0

532

79

275

0

0

0

5170

0

8

0

139

0

0

0

0

0

23

6

6

469

142

0

0

31

1364

988

0

1523

0

0

19

452

0

0

0

0

0

166

0

11

6

149

34

34

34

34

0

0

0

0

0

2602

210

0

0

1

1

0

15

15

0

0

0

14

14

0

180

23

19

138

0

0

0

343

71

0

71

135

5

26

8

0

1

94

1

0

137

137

0

0

0

0

0

0

0

0

0

0

0

0

0

0

0

498

0

0

15

15

0

0

0

6

6

0

193

193

0

39

39

0

0

0

0

0

0

0

194

11

82

73

2

0

0

26

0

0

51

0

10

7

25

0

0

0

0

0

7

0

2

0

0

230

0

0

15

15

169

169

0

0

46

46

0

0

0

0

0

0

0

0

0

0

0

0

108

6

1

0

5

0

0

0

71

71

31

0

24

1

6

58

58

57

1

0

0

0

198

1

1

0

0

0

61

61

0

0

0

0

0

2

2

20

20

0

0

114

17

97

1

1

0

1

956

439

439

0

0

12

12

14

14

0

0

0

0

430

30

0

0

0

92

25

184

29

18

15

32

5

0

0

12

12

34

0

34

0

0

0

0

0

0

0

0

15

15

1

1

0

0

1

1

216

0

0

0

0

0

216

80

6

1

55

18

35

11

24

30

30

0

0

0

20

20

51

18

33

0

15

15

15

15

17

17

17

0

0

17

0

0

0

0

0

0

0

0

0

0

0

0

0

0

11

11

11

11

63

63

63

63

0

0

0

0

0

0

0

0

0

0

0

0

0

0

0

0

0

0

0

236

236

0

0

17

0

17

14

14

53

17

19

0

0

0

17

0

0

1

1

66

48

18

0

0

0

85

33

18

34

0

0

0

0

0

0

0

0

323

27

27

27

0

0

0

0

0

0

0

0

0

172

172

172

0

0

0

0

0

0

0

0

0

124

124

124

1046

1046

0

0

33

33

0

0

0

0

85

69

16

0

0

0

0

0

0

0

0

0

0

0

0

1

1

40

40

0

0

0

0

0

3

1

0

0

1

1

867

25

372

470

0

0

0

0

17

0

17

0

7828

0

0

0

0

0

25

25

0

0

25

25

0

1

1

1

1

0

0

0

5212

1

0

0

1

1

78

0

0

0

0

78

78

38

0

0

0

0

0

38

38

0

0

0

0

0

0

0

0

0

0

162

162

162

0

0

2549

0

0

0

0

1315

1315

335

22

6

65

242

899

0

46

0

11

80

462

34

42

0

56

25

1

75

3

0

64

146

0

0

1

1

100

0

100

0

15

15

0

0

30

0

30

3

3

3

76

23

23

0

0

0

0

53

17

0

36

0

0

0

0

0

0

1

0

0

0

0

0

0

0

1

0

1

0

88

88

88

2061

0

0

12

0

0

12

10

10

13

13

30

30

89

81

0

8

642

150

492

8

8

106

0

106

0

0

100

0

100

119

99

20

0

0

0

52

0

52

880

880

0

0

9

0

0

9

0

9

51

29

29

28

1

0

0

0

0

22

22

0

22

0

0

0

0

0

0

0

0

21

21

21

21

0

0

0

0

0

0

0

0

0

0

0

0

0

0

0

0

0

0

0

0

0

0

0

0

0

0

0

89

0

0

0

8

2

2

0

6

6

0

0

0

0

0

0

0

0

0

0

0

0

0

0

0

0

0

0

0

0

0

0

0

0

0

0

0

0

0

0

53

53

0

1

49

3

28

0

0

0

0

28

28

0

0

0

0

0

0

0

0

0

0

0

0

0

0

0

0

0

0

0

0

0

0

0

0

0

0

0

0

0

0

0

0

0

0

0

1

0

0

0

1

1

1

0

0

0

0

0

0

0

0

0

73

2

0

0

2

2

2

2

2

3

0

0

0

3

3

0

0

0

55

29

29

10

10

16

16

0

0

3

3

0

3

0

0

0

0

0

0

3

3

3

5

0

0

3

3

0

0

0

0

1

0

0

0

1

1

1

0

0

0

0

0

0

0

0

0

0

0

0

21

21

21

0

0

0

0

21

0

0

0

0

0

0

0

0

0

0

0

0

0

0

2243

490

166

166

2

0

2

322

322

0

0

0

0

0

1753

27

27

0

0

0

0

34

34

0

14

0

4

10

0

1678

0

0

567

7

4

14

536

93

1

16

0

26

414

0

0

0

0

0

0

0

0

0

0

0

0

0

0

0

0

0

0

0

0

0

0

22

22

21

0

1

20

0

0

0

0

0

0

0

0

1

0

0

1

0

0

0

0

0

0

0

0

0

0

56

0

0

0

0

0

0

56

56

56

0

0

0

0

0

0

0

13

13

13

13

0

472

315

315

18

18

18

297

28

28

1

1

25

25

59

59

82

36

46

18

18

0

0

84

24

58

2

157

0

0

0

0

0

0

0

0

0

0

0

0

0

0

0

0

0

129

45

20

0

20

0

0

0

1

1

0

24

23

0

1

0

0

0

0

0

0

0

65

10

10

0

55

9

0

46

0

19

0

0

19

19

0

0

0

0

27

27

27

0

0

0

27

0

0

0

0

0

0

0

0

0

0

0

0

0

0

0

1

1

1

1

0

0

149

149

149

149

149

0

64

32

53

0

0

0

0

0

0

6410

0

0

0

0

0

6378

1078

3

3

3

1020

48

48

0

1

0

0

1

43

0

43

0

0

10

0

1

9

0

0

0

0

295

0

0

21

0

6

252

0

0

16

197

0

113

69

15

0

15

15

0

0

21

14

0

7

72

72

0

0

164

61

103

0

154

154

55

55

0

0

55

0

0

0

24

0

0

0

15

0

0

0

0

0

0

0

0

1

1

0

0

0

0

0

0

0

0

14

14

0

0

0

0

0

0

0

0

0

0

0

0

0

0

0

0

0

0

0

0

0

0

0

0

0

0

0

0

0

0

0

0

0

0

9

0

0

0

0

9

9

0

0

4768

0

0

0

0

0

0

0

0

0

0

0

83

3

3

3

3

4

4

73

73

8

1

0

1

0

0

0

0

0

0

0

0

2

2

5

0

0

2

0

0

0

0

3

0

0

52

52

52

3

0

0

0

0

3

0

1

2

0

0

0

0

0

0

0

0

40

40

0

0

0

11

17

0

0

0

0

0

7

0

1

0

1

0

3

292

3

3

275

275

0

14

6

3

3

1

0

1

1256

1

1

0

30

8

22

367

0

1

56

310

0

8

8

0

0

0

0

693

0

0

29

0

269

0

0

0

0

0

36

205

0

0

0

0

154

157

0

0

4

30

0

0

0

0

0

3

56

53

0

0

11

0

0

0

0

0

0

0

2965

2965

0

692

0

2273

0

0

0

0

0

69

69

69

85

0

0

0

33

0

0

0

0

33

33

0

0

52

14

14

0

0

0

0

38

10

28

0

0

0

423

127

0

0

0

0

12

12

115

32

48

35

0

0

0

0

0

0

0

0

0

0

0

2

0

0

2

2

0

0

0

0

0

0

0

0

0

0

0

26

23

23

0

0

3

0

3

0

268

1

1

154

7

20

0

94

0

33

0

0

0

0

0

1

0

1

112

12

23

0

0

31

1

3

12

1

4

17

0

8

0

0

0

0

0

16

16

16

0

0

16

16

3

3

0

0

0

3

3

3

13

13

13

13

0

13

0

0

0

0

0

0

0

0

0

344

212

0

0

0

0

0

0

0

0

0

0

0

0

0

0

0

0

0

0

0

0

0

0

0

0

0

0

0

212

212

212

212

0

0

0

0

0

0

0

132

132

132

74

50

24

54

54

4

0

4

0

0

0

0

0

0

0

0

0

0

0

0

8987

161

161

2

2

2

132

79

63

0

1

15

53

0

0

53

0

27

0

0

16

0

1

1

14

1

1

1

1

1

1

0

0

0

1

1

0

1

0

1

0

2

0

1

1

4

4

70

70

70

70

70

1466

69

45

0

0

0

0

34

34

0

0

2

2

9

9

0

0

24

24

0

24

215

49

49

49

4

4

4

4

4

4

0

0

0

0

0

0

158

158

158

309

12

12

12

0

0

0

81

81

81

7

7

7

195

195

195

14

14

14

7

7

7

7

866

46

4

2

2

42

0

42

0

0

0

0

242

242

1

15

226

262

35

0

35

197

0

197

30

30

0

316

0

0

0

0

316

316

2252

1267

0

0

0

742

569

0

89

1

1

212

157

109

0

0

0

0

0

91

0

11

80

60

36

9

15

22

22

525

82

82

0

0

443

443

0

0

0

0

985

343

343

23

319

1

642

0

0

1

1

641

641

4985

0

0

0

0

0

0

0

0

0

0

0

0

8

8

8

8

10

10

10

10

61

61

2

2

0

0

0

0

0

0

0

0

0

0

0

0

0

0

0

0

9

6

3

0

0

0

0

0

50

50

0

0

32

32

28

1

7

20

0

0

4

4

21

0

0

0

0

0

0

0

0

13

13

1

2

4

6

8

3

1

2

3

3

0

0

2

2

40

40

0

0

0

40

1

21

2

0

1

2

0

13

0

0

270

123

38

38

83

83

2

2

30

5

5

25

25

0

0

0

0

0

0

0

0

0

0

0

117

14

14

19

19

84

84

14

6

0

0

0

6

6

8

0

0

8

0

8

93

93

1

1

0

0

92

2

2

2

14

0

21

0

0

20

1

0

30

0

17

7

7

7

10

10

10

60

60

9

9

51

9

0

7

0

31

4

0

499

80

3

3

0

0

2

2

0

0

0

0

0

0

0

1

1

0

72

0

72

0

2

2

419

28

11

0

17

0

0

366

0

0

9

1

0

1

132

15

13

2

15

19

3

0

62

2

7

15

5

48

0

14

3

0

0

25

0

25

0

0

40

40

0

0

6

6

0

0

0

0

0

0

0

14

0

9

0

1

4

0

16

2

0

6

8

0

0

0

0

4

4

3820

3318

0

0

0

0

403

0

403

0

0

1

1

0

2462

0

1642

10

559

251

0

0

7

7

445

445

30

0

0

25

25

0

0

4

0

4

1

0

1

0

0

0

0

8

8

8

313

258

258

0

0

55

55

118

2

2

0

0

23

23

5

5

0

0

0

0

0

0

0

0

0

0

0

17

17

0

0

10

10

49

1

1

47

12

12

0

0

0

0

0

0

7

7

7

26

2

2

24

24

53

53

53

53

0

5

48

0

1588

1588

1588

1588

0

0

1587

57

0

0

46

0

574

60

0

42

87

586

0

0

0

0

0

0

0

0

135

0

0

0

1

1

0

0

0

0

0

0

0

0

0

0

0

0

616

616

616

616

616

616

1661

1661

1661

1616

0

0

0

0

0

0

1616

1615

1

45

45

0

0

0

0

0

0

45

0

0

575

575

575

7

7

0

0

7

0

0

568

568

268

300

0

0

0

0

0

0

0

0

0

0

41

0

0

0

0

0

17

17

8

8

8

9

9

9

0

0

24

24

0

0

0

1

1

1

23

23

23

26

26

26

26

26

19

7

0

0

610

226

13

13

13

13

213

79

70

70

0

9

9

0

0

0

0

134

44

44

1

1

0

0

89

89

137

21

21

21

6

15

0

0

0

0

0

0

0

0

0

116

116

0

0

17

0

11

6

28

0

28

51

19

0

32

20

0

0

20

17

0

0

0

0

17

17

17

17

0

0

0

0

85

85

85

0

0

85

21

1

0

0

20

1

18

24

0

0

0

0

0

145

145

16

16

16

129

129

129

0

0

0

0

0

0

0

1620

10

0

0

0

0

0

0

0

0

10

10

10

10

36

0

0

0

0

24

0

0

0

24

24

24

0

0

0

0

0

0

0

0

0

0

0

0

0

0

0

12

12

12

12

31

0

0

0

0

0

0

0

0

31

0

0

0

18

18

18

13

13

13

0

0

0

0

0

1543

1543

1056

1056

1056

34

34

34

4

4

4

87

87

87

0

0

7

7

7

163

0

0

142

0

142

0

0

0

0

0

0

21

21

21

21

21

171

171

171

16

16

16

0

0

0

16

16

16

0

0

0

0

0

0

764

764

764

764

0

0

652

143

509

0

0

0

112

112

209

169

31

31

19

19

12

12

0

0

0

0

0

138

40

40

40

69

69

69

0

0

0

0

0

0

16

16

16

13

13

13

0

0

0

0

0

40

14

14

9

9

5

0

5

26

26

26

0

26

85

85

85

10

10

10

0

0

75

16

16

0

59

0

5

0

0

0

3

51

0

0

0

0

0

0

0

0

13636

10317

1

1

0

0

0

0

0

1

0

0

0

1

0

0

11

11

11

11

0

0

1

0

0

0

0

0

0

1

1

1

0

0

7

7

7

0

6

1

1537

1374

2

2

0

0

76

32

0

0

44

90

29

61

0

0

0

48

48

0

0

0

0

2

1

1

1

1

0

0

0

0

0

0

0

0

9

2

7

0

9

2

0

7

0

0

16

1

8

1

0

0

0

0

0

0

4

1

0

1

0

0

0

0

0

0

0

0

0

0

0

21

0

21

0

0

44

3

39

2

40

13

4

0

22

0

1

31

0

0

0

0

29

0

1

1

0

1

1

41

0

0

1

39

1

92

92

0

0

0

0

0

0

0

4

4

0

0

0

0

0

9

9

3

3

69

0

11

55

3

0

0

0

0

126

126

3

3

0

0

0

0

0

0

0

0

46

7

39

0

0

0

463

0

258

4

31

0

93

30

2

0

1

1

43

128

128

0

0

0

0

163

163

30

133

8475

4941

295

295

2900

0

90

2810

231

231

0

8

8

781

781

0

0

0

724

568

31

123

0

2

2

2

0

0

0

1664

1

1

22

22

1423

1423

0

0

177

177

0

0

41

41

0

1870

0

0

0

0

0

0

0

0

0

915

915

955

955

30

30

21

21

0

0

9

4

2

1

0

1

0

1

0

0

38

26

26

26

0

0

0

0

0

0

0

0

0

12

12

12

0

0

0

0

0

0

0

0

0

159

119

0

0

0

0

0

6

6

0

0

0

0

0

0

0

6

0

6

0

0

0

0

0

0

1

1

0

0

0

35

1

1

0

29

4

5

0

1

0

0

1

0

2

1

0

0

0

0

28

28

31

12

19

7

0

0

0

7

9

9

9

0

0

0

7

0

0

0

7

7

0

0

0

0

0

0

0

0

0

0

0

0

0

0

0

0

24

18

0

0

0

0

5

13

2

2

0

0

0

0

0

0

0

0

0

2

0

0

1

1

2

2

0

0

0

0

0

0

0

0

0

58

27

27

27

0

0

0

1

1

1

30

30

30

0

0

0

2814

0

0

0

0

947

281

0

0

0

0

0

0

2

0

2

0

0

0

13

0

13

178

178

0

0

58

58

30

30

666

0

0

0

0

0

0

0

666

3

0

2

45

1

16

134

2

0

48

385

7

1

0

0

18

4

0

0

0

0

28

28

8

8

0

20

20

0

0

256

112

0

0

0

0

43

43

14

4

0

0

0

9

1

46

46

0

0

6

4

2

3

3

144

27

27

19

19

9

3

6

1

0

1

52

47

5

0

0

0

0

6

6

14

1

13

3

3

11

11

2

2

0

0

0

0

0

0

0

0

0

0

0

0

92

92

2

2

0

0

90

3

87

0

0

0

0

0

7

7

7

2

5

630

630

0

0

0

0

458

28

201

0

4

2

0

2

0

1

0

32

0

0

0

112

0

0

35

0

1

0

32

0

0

0

8

44

44

44

44

84

0

54

30

1

0

0

0

0

0

0

0

0

0

1

1

1

0

0

0

0

0

718

8

8

6

2

1

0

0

0

0

1

1

0

0

0

0

158

148

0

4

5

0

2

0

31

32

69

0

0

5

0

10

8

2

2

2

0

2

547

1

1

1

1

8

2

4

2

3

0

1

2

289

0

91

96

1

0

101

4

4

93

92

0

1

0

0

0

13

13

109

0

1

108

2

2

0

12

12

12

2

10

0

0

0

2

0

0

0

0

2

2

130

130

130

0

0

126

0

0

4

5

5

0

0

0

0

5

5

27

27

27

27

27

0

0

0

0

0

0

0

0

0

0

459

0

0

0

0

0

0

459

0

0

0

94

0

0

0

0

0

0

0

0

0

94

78

0

0

0

11

5

0

0

0

365

0

0

0

20

20

345

4

192

149

0

0

0

0

0

0

0

19

19

19

19

19

134

43

22

18

18

18

4

4

4

0

0

17

17

17

17

4

2

2

2

0

0

0

2

2

2

1

1

1

1

1

0

0

0

0

0

0

0

0

0

0

0

34

5

5

5

5

15

15

0

0

0

0

0

0

15

15

7

7

7

7

0

0

0

1

1

0

0

1

1

0

0

6

0

0

0

3

3

3

3

3

3

56

56

56

56

56

0

0

0

0

0

0

0

225

225

225

225

225

130

95

0

0

0

0

0

0

57

57

57

0

0

0

0

0

0

3

3

3

54

54

54

0

0

0

0

0

0

1

1

1

1

1

1

1

0

0

0

0

0

0

0

0

0

0

0

0

0

0

0

0

0

0

0

0

0

0

0

0

0

0

0

0

0

0
